# Two Metabolomics Phenotypes of Human Hepatocellular Carcinoma in Non-Alcoholic Fatty Liver Disease According to Fibrosis Severity

**DOI:** 10.3390/metabo11010054

**Published:** 2021-01-14

**Authors:** Benjamin Buchard, Camille Teilhet, Natali Abeywickrama Samarakoon, Sylvie Massoulier, Juliette Joubert-Zakeyh, Corinne Blouin, Christelle Reynes, Robert Sabatier, Anne-Sophie Biesse-Martin, Marie-Paule Vasson, Armando Abergel, Aicha Demidem

**Affiliations:** 1 Department of Digestive and Hepatobiliary Medecine, CHU Clermont-Ferrand, F-63000 Clermont-Ferrand, France; bbuchard@chu-clermontferrand.fr (B.B.); camille.teilhet@hotmail.fr (C.T.); smassoulier@chu-clermontferrand.fr (S.M.); aabergel@chu-clermontferrand.fr (A.A.); 2 INRA, Human Nutrition Unit, Clermont Auvergne University, F-63000 Clermont-Ferrand, France; nataliabeywickramasamarakoon@gmail.com (N.A.S.); m-paule.vasson@uca.fr (M.-P.V.); 3 Department of Anatomo-pathology, CHU Clermont Ferrand, F-63000 Clermont-Ferrand, France; jjoubert@chu-clermontferrand.fr (J.J.-Z.); cblouin@chu-clermontferrand.fr (C.B.); 4 Laboratory of Biostatistics, Computer Science and Pharmaceutical Physics, UMR 5203, Faculty of Pharmacy, F-34 093 Montpellier, France; christelle.reynes@umontpellier.fr (C.R.); robert.sabatier@umontpellier.fr (R.S.); 5 Team RMN-START, Clermont Auvergne University, F-63000 Clermont-Ferrand, France; a-sophie.biesse-martin@uca.fr; 6 UMR CNRS 6284, Clermont Auvergne University, F-63000 Clermont-Ferrand, France

**Keywords:** hepatocarcinoma, fibrosis, non-alcoholic fatty liver disease, nuclear magnetic resonance, metabolomics

## Abstract

Non-Alcoholic Fatty Liver Disease (NAFLD) is considered as the forthcoming predominant cause for hepatocellular carcinoma (HCC). NAFLD-HCC may rise in non-cirrhotic livers in 40 to 50% of patients. The aim of this study was to identify different metabolic pathways of HCC according to fibrosis level (F0F1 vs. F3F4). A non-targeted metabolomics strategy was applied. We analyzed 52 pairs of human HCC and adjacent non-tumoral tissues which included 26 HCC developed in severe fibrosis or cirrhosis (F3F4) and 26 in no or mild fibrosis (F0F1). Tissue extracts were analyzed using ^1^H-Nuclear Magnetic Resonance spectroscopy. An optimization evolutionary method based on genetic algorithm was used to identify discriminant metabolites. We identified 34 metabolites differentiating the two groups of NAFLD-HCC according to fibrosis level, allowing us to propose two metabolomics phenotypes of NAFLD-HCC. We showed that HCC-F0F1 mainly overexpressed choline derivatives and glutamine, whereas HCC-F3F4 were characterized by a decreased content of monounsaturated fatty acids (FA), an increase of saturated FA and an accumulation of branched amino acids. Comparing HCC-F0F1 and HCC-F3F4, differential expression levels of glucose, choline derivatives and phosphoethanolamine, monounsaturated FA, triacylglycerides were identified as specific signatures. Our metabolomics analysis of HCC tissues revealed for the first time two phenotypes of HCC developed in NAFLD according to fibrosis level. This study highlighted the impact of the underlying liver disease on metabolic reprogramming of the tumor.

## 1. Introduction

Hepatocellular carcinoma (HCC) is a concerning disease worldwide as it is the sixth most common malignant tumor and the third leading cause of cancer death [1]. For the past 20 years, the increasing incidence of HCC is suspected to be related to the increasing burden of Non-Alcoholic Fatty Liver Disease (NAFLD) [2]. Notably, it was reported that around ten percent of HCCs rise on a non-cirrhotic background, including chronic hepatitis B and C infections, hemochromatosis, and NAFLD [3].

NAFLD is closely linked to abdominal obesity, hypertension, dyslipidemia, and type 2 diabetes through insulin resistance and impaired glucose metabolism. It is considered as the liver manifestation of the metabolic syndrome (MS) [4]. It covers a wide spectrum of various chronic liver diseases: simple steatosis, steato-hepatitis, and all-grade fibrosis. The METAVIR classification is currently the reference for assessing histological fibrosis level from absence of fibrosis (F0) to cirrhosis (F4) [5]. NAFLD has become the most common liver disorder in industrialized countries, affecting up to 25% of the adult population in western countries [2]. Recently, it was proposed to revise the fatty liver nomenclature, NAFLD being substituted for Metabolic Associated Fatty Liver Disease (MAFLD), by including anthropometric and metabolic phenotyping approaches [4].

Forty to 50% of NAFLD associated HCC (NAFLD-HCC) have no evidence of cirrhosis [6,7]. A French cohort of HCC in patients with MS showed that these patients were more often free of severe fibrosis (F0–F2), at odds with the paradigm that severe fibrosis (F3–F4) is a necessary step in carcinogenesis and implying different carcinogenic pathways [8]. Moreover, a recent review underlined that hepatocarcinogenesis could be promoted by both fibrosis dependent and independent mechanisms [9]. NAFLD-HCC is detected more frequently at a later tumor stage as these patients escape surveillance [7]. Histological patterns indicate a less aggressive phenotype with fewer microvascular invasion and satellite nodules [10]. These tumors are more often well-differentiated [8]. Pro-carcinogenic factors for NAFLD-HCC have been well defined in the last years, including insulin resistance and PNPLA3 polymorphism [11]. Despite growing evidence, the population with NAFLD at the highest risk of developing HCC is underdiagnosed. Currently, international guidelines do not propose a dedicated surveillance for these patients apart from those with cirrhosis [12]. Considering the large and increasing number of patients with NAFLD, early detection remains a major challenge.

So far, understanding the pathophysiology of NAFLD-HCC has not given the key for the identification of high-risk patients. Multiple mechanisms have been established in NAFLD-HCC, including hyperinsulinemia, proinflammatory pathways, abnormal adipokines secretion, oxidative stress, epigenetic alterations, and mobilization of hepatic progenitor cells [11]. Putative pathways linking fibrosis level and NAFLD-HCC, particularly regarding metabolic signatures, have not been thoroughly investigated. Thus, the identification of metabolic pathways could lead to the development of relevant screening and diagnosis tools. This approach may provide appropriate treatments targeting specific pathways in NAFLD-HCC. We recently reported specificities of human HCC developed on non-cirrhotic NAFLD vs. HCC associated with cirrhosis of various etiologies [13]. More investigations are needed to identify specific carcinogenic pathways involved in HCC developed exclusively in NAFLD according to the fibrosis status.

Metabolomics is being widely used to get insights into carcinogenesis mechanisms. Most metabolomics studies of HCC were based on animal models or applied to serum and urine of patients, using mass spectrometry or Nuclear Magnetic Resonance (NMR) spectroscopy [14]. At present, few metabolomics studies have investigated HCC using human tissues [15]. Moreover, metabolomics studies focusing specifically on NAFLD-HCC are scarce. To our knowledge, there are no reports concerning the impact of fibrosis severity on the metabolic profile of NAFLD-HCC. To gain insight into the mechanisms of the underlying disease, we performed a metabolomics tissue analysis using proton NMR spectroscopy.

We speculated that the degree of liver fibrosis might interfere with metabolomics profiles of tumors. Thus, according to the severity of fibrosis, NAFLD-HCC might present specific metabolic phenotypes suggesting different signaling pathways and distinct mechanisms of carcinogenesis. The aim of our study was to investigate firstly the metabolomics profile of tumoral and non-tumoral tissue from NAFLD-HCC patients according the stage of fibrosis (F3F4) versus (F0F1) and secondly to identify specific tissue metabolic signatures.

A total of 52 pairs of matched HCC tissues and distal non-tumoral tissues (NTT) (taken at 2 cm from tumor localization) were assessed using ^1^H-NMR-based metabolomics to explore direct metabolic changes in the liver. Metabolomics analyses underlined several biochemical alterations involving glycolysis, TCA cycle, oxidative response, transmethylation reactions, lipids and phospholipids metabolisms. Based on the analysis of extract tissues, we report 34 metabolites with differences in abundance in two groups of NAFLD-HCC according to fibrosis status (F0F1 versus F3F4), allowing us to propose two specific metabolomics phenotypes of NAFLD-HCC.

## 2. Results

### 2.1. Patients Characteristics

Our HCC cohort (*n* = 52) included 45 males and 7 females with a mean age of 70 years. Clinical, biological, histological features of the two groups are reported in Table 1.

There were no differences between the two groups regarding body mass index, diabetes status or preoperative AFP level. There was no difference of steatosis severity between the two groups of NAFLD-HCC. Notably, severe steatosis (>33%) was still found in almost 20% of patients with severe fibrosis.

Despite a higher tumor burden, recurrence free survival and overall survival did not differ between the two groups of HCC (Supplementary Figures S1 and S2A,B). Indeed, NAFLD-HCC is more often detected at a later tumor stage [7]. However, the absence of severe fibrosis resulting in more favorable outcomes after surgery and the lower incidence of HCC in patients with non-cirrhotic NAFLD probably explain the similar prognosis observed between the two groups of NAFLD-HCC [3].

### 2.2. Identification of Discriminant Metabolites

Table 2 provides the list of discriminating metabolites with their respective chemical shift (ppm) between the two groups of tissues (aqueous and lipid extracts). Most of the metabolites belonged to glucose metabolism, Krebs cycle, energy metabolism, amino acids (AA), phospholipids (Plp), and lipids metabolism.

### 2.3. Differential Metabolites between NAFLD-HCC-F0F1 vs. NTT-F0F1

**Figure 1:**
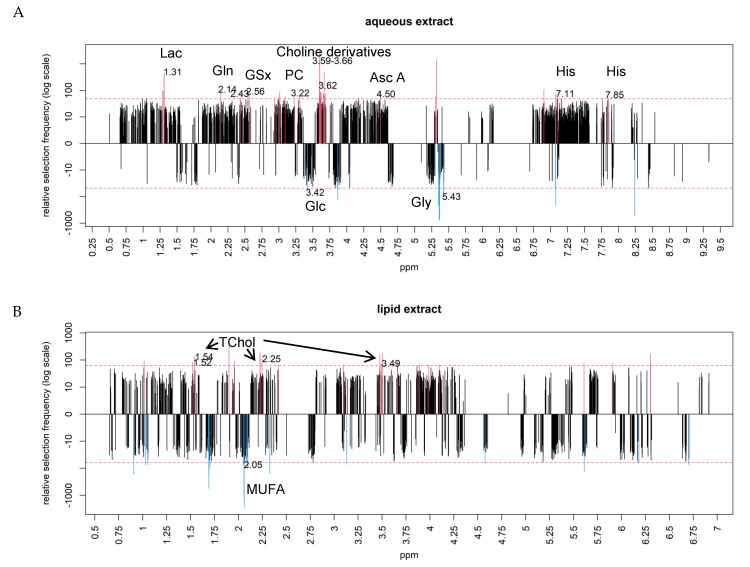

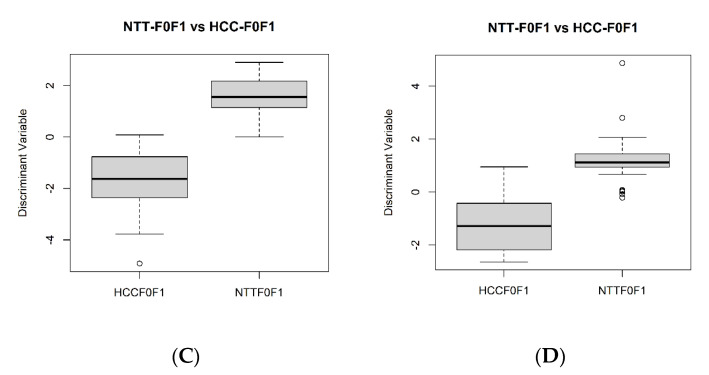
Frequency of selection of each ppm location in the aqueous (**A**) and lipid phases (**B**) from the comparison between hepatocellular carcinoma (HCC)-F0F1 and non-tumoral tissues (NTT)-F0F1. Discriminant metabolites (above the number of selection threshold in dotted line) are illustrated in red for upregulated and blue for downregulated metabolites in HCC. In the aqueous phase (**A**), lactate (ppm 1.31), glutamine (ppm 2.14/2.43), glutathione (ppm 2.56), phosphocholine (ppm 3.22), choline derivatives (ppm 3.59–3.66), ascorbic acid (ppm 4.50), histidine (ppms 7.11/7.85), glucose (ppm 3.42), and glycogen (ppm 5.43) were identified as discriminant metabolites. In the lipid phase (**B**), total cholesterol (ppm 1.52–1.54/2.25/3.49) and monounsaturated fatty acids (ppm 2.05) were identified as discriminant metabolites. Subsets of metabolites in the aqueous (**C**) and lipid phases (**D**) demonstrated significant accuracy to differentiate HCC from NTT in F0F1 fibrosis.

First, our metabolomics results showed that glucose (Glc) level decreased in HCC, while lactate (Lac) level increased. This universal observation known as the “Warburg effect” is a major biochemical trait of tumor cells. Second, in NAFLD-HCC F0F1, the accumulation of glutamine (Gln) may be consistent with an activation of glutamine synthetase (GS) which converts glutamate to glutamine in pericentral hepatocytes. We also found an accumulation of Histidine (His) content. In addition, HCC displayed an increase of glutathione (GSx) and ascorbic acid (Asc A) contents that may correspond to a preserved anti-oxidant response in HCC-F0F1. A decrease of glycogen level was also observed. Third, the most relevant observation is that HCC exhibited an increase in choline derivatives, including phosphocholine (PC), a precursor and a breakdown product of phosphatidylcholine (PtdCho), suggesting a change in membrane structure and function with the possible activation of choline kinase (CK) and PtdCho hydrolysis involving phospholipases (Figure 1A).

Lipids extracts analysis indicated a decrease of monounsaturated fatty acids (MUFA) level and an increase in total cholesterol (TChol) in HCC compared to NTT which suggest a change in lipid metabolism (Figure 1B).

Supplementary Table S1 provides the list of discriminant metabolites according to the number of selections found in the comparison between NTT-F0F1 vs. HCC-F0F1.

These subsets of metabolites significantly discriminated NTT-F0F1 from HCC-F0F1 in both aqueous and lipid phases, as illustrated in Figure 1C,D, respectively. These metabolic signatures demonstrated very high areas under curve (AUC) of 0.98 and 0.94 (Supplementary Figure S3A,B).

### 2.4. Differential Metabolites between NAFLD-HCC-F3F4 vs. NTT- F3F4

**Figure 2:**
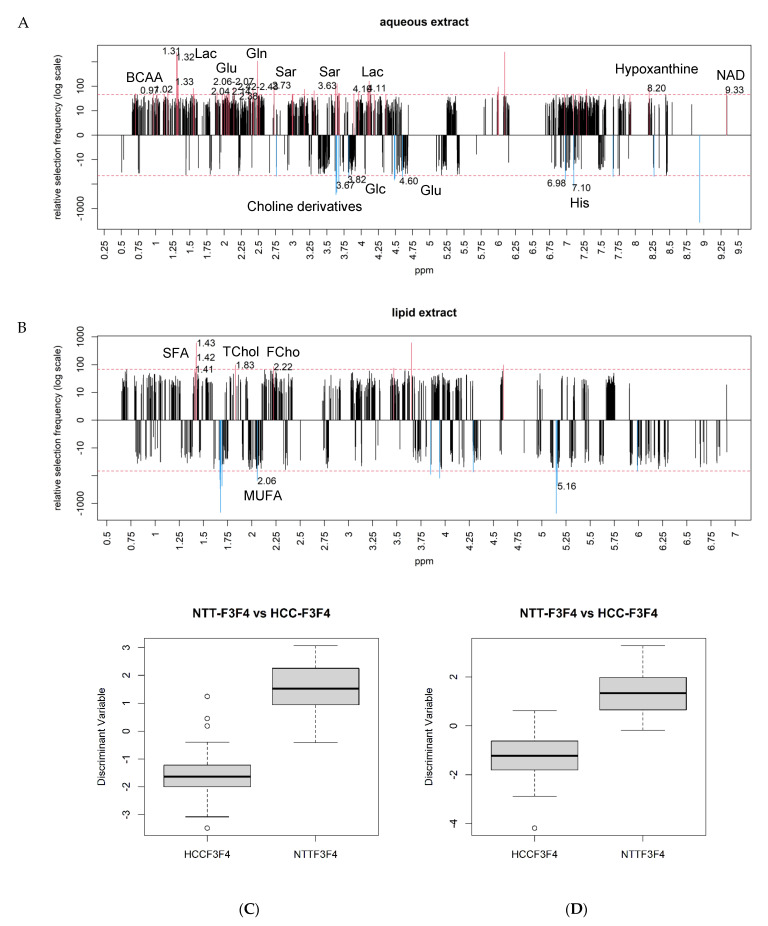
Frequency of selection of each ppm location in the aqueous (**A**) and lipid phases (**B**) from the comparison between HCC-F3F4 and NTT-F3F4. Discriminant metabolites (above the number of selection threshold in dotted line) are illustrated in red for upregulated and blue for downregulated metabolites in HCC. In the aqueous phase (**A**), Branched Chain Amino Acids (BCAA) (ppm 0.97/1.02), lactate (ppm 1.31–1.33/4.10–4.11), glutamine (ppm 2.14/2.42–2.43), glutamate (ppm 2.06–2.07/2.38), sarcosine (ppm 2.73/3.63), hypoxanthine (ppm 8.20), Nicotinamide Adenine Dinucleotide (NAD) (ppm 9.33), glucose (ppm 3.82/4.60), choline derivatives (ppm 3.67), and histidine (ppm 6.98/7.10) were identified as discriminant metabolites. In the lipid phase (**B**), saturated fatty acids (ppm 1.41–1.43), total cholesterol (ppm 1.83), free cholesterol (ppm 2.22), and monounsaturated fatty acids (ppm 2.06) were identified as discriminant metabolites. Subsets of metabolites in the aqueous (**C**) and lipid phases (**D**) demonstrated significant accuracy to differentiate HCC from NTT in F0F1 fibrosis.

HCC metabolomics profile displayed the hallmark of “Warburg effect” with an increase of Lac level and a decrease of Glc level. HCCs exhibited an increase of Gln and Glu. These results could indicate a decrease of TCA cycle activity in tumors, an impaired glutaminolysis or an alteration of the urea cycle. Moreover, HCCs moderately accumulated Branched Chain Amino Acids (BCAA), such as Valine (Val), Leucine (Leu), and Isoleucine (IsoLeu), which may reflect an activation of mTORC1 pathway. The increased content of Sarcosine (Sar) and decreased level of His may imply methylation disorders in HCC developed in severe fibrosis. In addition, tumors exhibited an increased level of both *Nicotinamide Adenine Dinucleotide* (NAD) and hypoxanthine levels, metabolites which are required for tumor cell proliferation and survival (Figure 2A).

Lipid analysis indicated a significant increase of saturated fatty acids (SFA) and decrease of MUFA in HCC compared to NTT. These data may indicate abnormal activities of enzymes involved in de novo lipogenesis (DNL), such as Fatty Acid Synthase (FASN) and stearoyl-coA desaturase (SCD). Meanwhile, an accumulation of free cholesterol (FChol) and TChol was found which could be in favor of mTOR activation, consistent with the increase in BCAA found in the aqueous phase (Figure 2B).

Supplementary Table S2 provides the list of discriminant metabolites according to the number of selections found in the comparison between NTT-F3F4 vs. HCC-F3F4.

These subsets of metabolites significantly discriminated NTT-F3F4 from HCC-F3F4 in both aqueous and lipid phases as illustrated in Figure 2C,D, respectively. These metabolic signatures demonstrated very high AUC of 0.97 and 0.98 (Supplementary Figure S4A,B).

### 2.5. Differential Metabolites between NAFLD-HCCs according the Severity of Fibrosis

**Figure 3:**
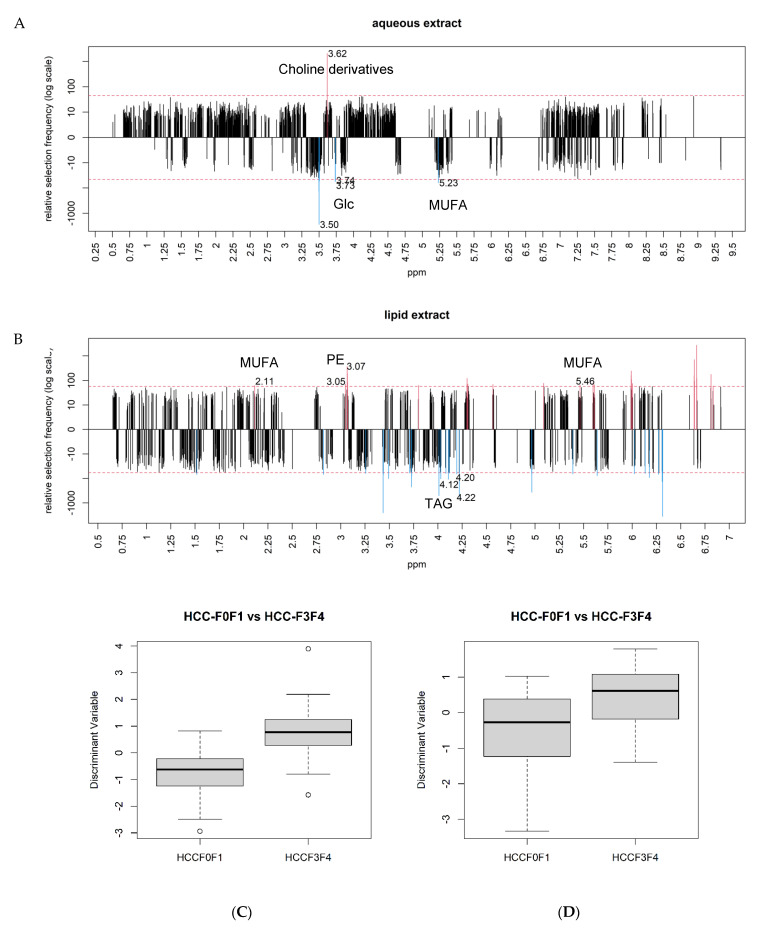
Frequency of selection of each ppm location in the aqueous (**A**) and lipid phases (**B**) from the comparison between HCC-F0F1 and HCC-F3F4. Discriminant metabolites (above the number of selection threshold in dotted line) are illustrated in red for upregulated and blue for downregulated metabolites in HCC-F0F1. In the aqueous phase (**A**), choline derivatives (ppm 3.62), glucose (ppm 3.50/3.73–3.74), and monounsaturated FA (ppm 5.23) were identified as discriminant metabolites. In the lipid phase (**B**), monounsaturated fatty acids (ppm 2.11/5.46), phosphoethanolamine (ppm 3.05–3.07), and triacylglycerides (ppm 4.12/4.22) were identified as discriminant metabolites. Subsets of metabolites in the aqueous (**C**) and lipid phases (**D**) demonstrated significant accuracy to differentiate HCC from NTT in F0F1 fibrosis.

The observed accumulation of Glc in HCC F3F4 could be a consequence of an enhanced neoglucogenesis process. Thus, the increase of Glc content may confirm the putative activation of mTOR pathway in severe fibrosis (Figure 3A). We found an increased level of choline derivatives (Cho/PC/PtdCho) in HCC-F0F1 vs. HCC F3F4, which is consistent with the observation in HCC-F0F1 reported in the comparison between HCC-F0F1 vs. NTT-F0F1 (Figure 3A). These data strongly suggest that these metabolites play a paramount role in this carcinogenesis process.

Other HCC metabolic signatures arose from lipid analysis. Low levels of MUFA and increased triacylglycerol (TAG) content in HCC-F3F4 suggested different lipid reprogramming according to fibrosis level. In addition, we found an increased content of phosphoethanolamine (PE) in HCC-F0F1 in accordance with the increase of choline derivatives in the aqueous phase (Figure 3B).

Supplementary Table S3 provides the list of discriminant metabolites according to the number of selections found in the comparison between HCC-F0F1 vs. HCC-F3F4.

These subsets of metabolites significantly discriminated HCC-F3F4 from HCC-F0F1 in both aqueous and lipid phases as illustrated in Figure 3C,D, respectively. These metabolic signatures demonstrated high AUC of 0.88 and 0.76 (Supplementary Figure S5A,B).

Genetic algorithm (GA) and metabolomics analysis, in the context of NAFLD, highlight, for the first time, that there are two phenotypes of HCC developed in NAFLD according to fibrosis level.

## 3. Discussion

To our knowledge, our study investigates for the first time tissue metabolome of human NAFLD-HCC according to the degree of fibrosis as the underlying pathology. Our work revealed major metabolic alterations in NAFLD-HCC concerning glycolysis, AA levels, methylated species, phospholipids derivatives, and lipids content.

### 3.1. Carbohydrate Metabolism in NAFLD-HCC: A Common Warburg Effect but Enhanced Neoglucogenesis in Severe Fibrosis

The comparison between HCC to NTT indicated that, irrespective of the degree of fibrosis, tumors displayed a decreased level of Glc and an increase of Lac content. This biological pattern of cancer known as the Warburg effect consists of enhanced glycolysis, low levels of oxidative phosphorylation and high Lac production are observed [16]. Previous works supported the notion of a favored glycolytic pathway in HCC through the upregulation of hexokinase, glyceraldehyde-3-phosphate dehydrogenase, and pyruvate kinase [16,17,18].

In addition, when comparing both groups of HCC, a significant accumulation of Glc in HCC developed on severe fibrosis was highlighted suggesting an enhanced neoglucogenesis process through an upregulation of lactate deshydrogenase (LDH) in a mTOR dependent manner. Positron-emission tomography (PET) scan using fluorodeoxyglucose (FDG) is currently used in HCC for tumor staging. Its use in clinical practice reinforces our data demonstrating strong Glc accumulation in tumors [19].

### 3.2. Preserved Antioxidant Defenses in HCC-F0F1

Our results indicated an increased level of major antioxidant agents that are GSx and ascorbic acid. Oxidative stress is a well-known driver of NAFLD progression with increased Reactive Oxygen Species (ROS) and decreased antioxidants levels [20]. It was suggested that ROS production could be a protective mechanism to induce an upregulation of antioxidant defenses in NALFD [21]. This hypothesis corroborates our findings suggesting a preserved production of antioxidant defenses in HCC tissue in the absence of fibrosis in response to an increased oxidative stress in NAFLD.

### 3.3. Enhanced Glutamine Synthetase Activity in HCC-F0F1 and Putative Involvement of the Beta-Catenin Pathway in NAFLD

Another noteworthy finding of our study is that HCC-F0F1 specifically exhibited a high level of Gln compared to NTT. Our previous study was the first one to reveal the increased levels of Gln in HCC-F0F1, which correlated with an overexpression of GS [13]. GS activity is impaired in chronic liver diseases and glutamine levels gradually decrease from healthy liver to established cirrhosis [22] as confirmed in our study (data not shown). GS immunostaining is detected in preneoplastic lesions and in HCC, preferentially in tumors developed on cirrhosis [22]. GS positive labeling is an important histological hallmark of HCC, including early HCC, and has been associated with poor prognosis following surgery [23,24]. The overexpression of GS is highly correlated with β-catenin mutation and GS is proposed as a reliable marker of β-catenin activation secondary to its mutation [25]. To our knowledge, there is no data in the literature on GS activity, especially in NAFLD-HCC, according to fibrosis severity. How the Gln metabolism and whether the beta-catenin pathway is specifically involved in NAFLD carcinogenesis remains unknown.

Comparing HCC-F3F4 and NTT-F3F4, our data revealed higher levels of both Gln and Glu in tumors. These results corroborate previous studies reporting that patients with HCC have decreased plasma and tissue levels of GSx, since Glu being an important component of GSx [26]. Based on a global transcriptomic analysis of multiple human HCC, Bjornson et al. identified a significant downregulation of enzymes involved in glutaminolysis in HCC [27]. Data from this study are in accordance with our previous published results which demonstrated that HCC developed on cirrhosis accumulated hydroxybutyrate, a metabolite which supplies the TCA (tricarboxylic acid) cycle with acetyl-coA [13].

### 3.4. BCAA Content and Possible Activation of the mTOR Pathway in HCC-F3F4

BCAAs are essential ketogenic amino acids. In chronic liver disease, serum concentrations of BCAAs decreased, while the concentrations of aromatic amino acids (AAA) increased [28]. It has also been reported that the ratio of BCAAs to AAAs called the Fischer ratio were lower in serum of patients with HCC compared to controls [28].

Our metabolomics data showed an increase in BCAA content (Val, Leu, IsoLeu) in HCC-F3F4 compared to NTT-F3F4, as previously reported in tissue metabolomics analysis [29,30]. Loss of BCAA mitochondrial catabolism was held responsible for BCAA accumulation in HCC and mTOR activation [29]. BCAA activate the mTOR pathway in a Rag GTPase manner depending on nutritional environment [31].

Previous reports highlighted a link between obesity-associated insulin resistance and increase of BCAA through a mTORC1 mechanism and impaired autophagy [31]. These data corroborate our findings in NAFLD-HCC in the presence of severe fibrosis.

From our data, we propose that NAFLD-HCC developed on severe fibrosis may have a higher affinity for BCAA. BCAA accumulation results from an impaired mitochondrial catabolism which activate mTOR pathway. This is consistent with other data indicating that mitochondria functions are impaired in NAFLD-HCC. Tanaka et al. quantified increased oxidative stress through elevated content of 8-Hydroxy-2′-Deoxyguanosine in NASH-HCC tissues compared to NASH without HCC and HCC of various etiologies [32]. However, the impact of fibrosis severity was not evaluated.

### 3.5. Methylation Disorders in HCC-F3F4

In NAFLD-HCC F3F4, the increase of Sar level, a methyl donor, suggests the involvement of the oncoprotein Glycine N-methyltransferase (GNMT). This enzyme plays a pivotal role in the biochemical conversion of Glycine (Gly) to Sar with the addition of one methyl group. GNMT is a key enzyme for methylation reactions and epigenetic modulation through DNA and histone methylation. Previous reports showed that GNMT expression was significantly downregulated in human HCC [33]. Sar (*N*-methylglycine) was previously delineated as a substantial oncometabolite of prostate cancer and its metabolism seems to be significantly involved in prostate cancer development [33]. We also observed in NTT-F3F4 an increase in glycine content compared to NTT-F0F1 (data not shown) which reinforces our hypothesis of methylation disorders in severe fibrosis, even in the peritumoral environment, and the notion of metabolic continuum between tumor and NTT.

Our data indicate that His levels decrease in HCC-F3F4. His metabolism is coupled with the folate cycle by its transformation into formiminoglutamate, which is also consistent with the increase of Glu found in HCC-F3F4 [34]. The formimino group is then transfered to THF (tetrahydrofolate) to form fomiminoTHF, a metabolic precursor of methyl THF involved in the synthesis of methionine, a major methyl donor [33].

### 3.6. NAFLD-HCC in Non-Severe Fibrosis Displays a Cholinic Phenotype

An important result of this study is that HCC developed on NAFLD without or with mild fibrosis exhibited first a significant increase of choline derivatives, including PC, compared to its non-tumor adjacent tissue and second an increase of PE when compared to HCC-F3F4. Our study is the first one to provide evidence that HCC without fibrosis exhibit a new Plp phenotype.

PC is an intermediate in the synthesis of PtdCho in tissues and a breakdown product of cell membrane [35]. PC was also shown to promote cell proliferation effects of insulin and insulin-like growth factor-1 [36]. Previous reports in human HCC and in other cancers highlighted an upregulation of CK consistent with a modification in cell membrane synthesis, structure and function [17,37]. Choline derivatives accumulation in cancer cells play a role in the stimulation of mitogenesis and in the maintainenance of PtdCho homeostasis, which is critical for cell survival. Activated choline metabolism, characterized by increased PC and choline derivatives compounds is referred to as a cholinic phenotype [35]. CK has also been incriminated in HCC by helping the interaction between Epidermal Growth Factor Receptor and mTORC2 [37]. Whether CK participates in hepatocarcinogenesis as an oncometabolite in cell signaling or as precursor of cell membrane compounds remains unclear.

This observation might provide a rationale for increased radiolabeled choline uptake on PET-scan for HCC developed in NAFLD without fibrosis. Interestingly, a correlation between ^18^F Fluorocholine uptake and the degree of fibrosis in the liver was recently reported in patients undergoing surgery for HCC: liver mean Standardized Uptake Value decreased as the stage of fibrosis increased [38]. More studies focusing on early detection and progression of NAFLD-HCC without fibrosis are urgently needed and PET scan with Fluorocholine is a promising tool.

PE is produced from Ethanolamine under the action of cytosolic Ethanolamine Kinase located specifically in the liver [39]. It belongs to the CDP (cytidine diphosphate) ethanolamine pathway ultimately leading to Phosphatidylethanolamine (PtdEth), an important component of inner cell membrane. This pathway parallel the CDP-choline pathway for PC synthesis, in agreement with the increase in PC found in HCC-F0F1. The balance between PtdCho and PtdEth is fundamental in the liver, any disturbance resulting in steatosis [39]. The involvement of Ethanolamine derivatives in cancer has been little investigated. PtdCho also derives from PtdEth under the action of Phosphatidylethanolamine N-methyltransferase (PEMT), consuming three methyl groups. In human HCC, a downregulation of PEMT has been reported [40]. However, the activity of PEMT according to fibrosis level needs to be addressed.

### 3.7. Different Lipid Metabolism Reprogramming in NAFLD-HCC according to Fibrosis Severity

Comparing the metabolic profile of HCC to NTT, independently of the severity of fibrosis, a lower level of MUFA was found in tumors. This result was unexpected since it was demonstrated that metabolic diseases, including NAFLD and HCC, display increased DNL [41,42]. We are aware that the expression of MUFA should be confirmed by a more specific method for lipids, such as mass spectroscopy. Cumulating evidence underlines aberrant lipid biosynthesis as an early and crucial event in carcinogenesis. Elevated MUFA content is a signature of many tumors, including HCC, and results from an enhanced activity of SCD1 [43]. The elevated expression of this enzyme is associated with poor prognosis and cancer aggressiveness [43]. The increased level of MUFA-PtdCho is a biochemical trait of HCC and could explain the decrease of MUFA in our tumor tissues analysis [44]. Otherwise, MUFA-PtdCho was shown to significantly correlate with proliferative fingerprint metabolites [45]. The lower level of MUFA observed in HCC-F3F4 compared to HCC-F0F1 may be associated with a higher proliferative rate in presence of severe fibrosis. This hypothesis is consistent with the increased content of NAD and hypoxanthine found in HCC-F3F4.

We also highlighted an increased content of SFA in HCC-F3F4 compared to its NTT. It was recently demonstrated that some HCCs, particularly beta-catenin driven tumors, are not dependent on FASN activity and DNL for their growth [46]. Thus, we hypothesize that some tumors rise under the influence of the beta-catenin pathway and, therefore, may be less dependent of DNL, such as HCC-F0F1.These NMR metabolomics data should be confirmed by immunohistochemistry and transcriptomic analysis.

**Figure 4:**
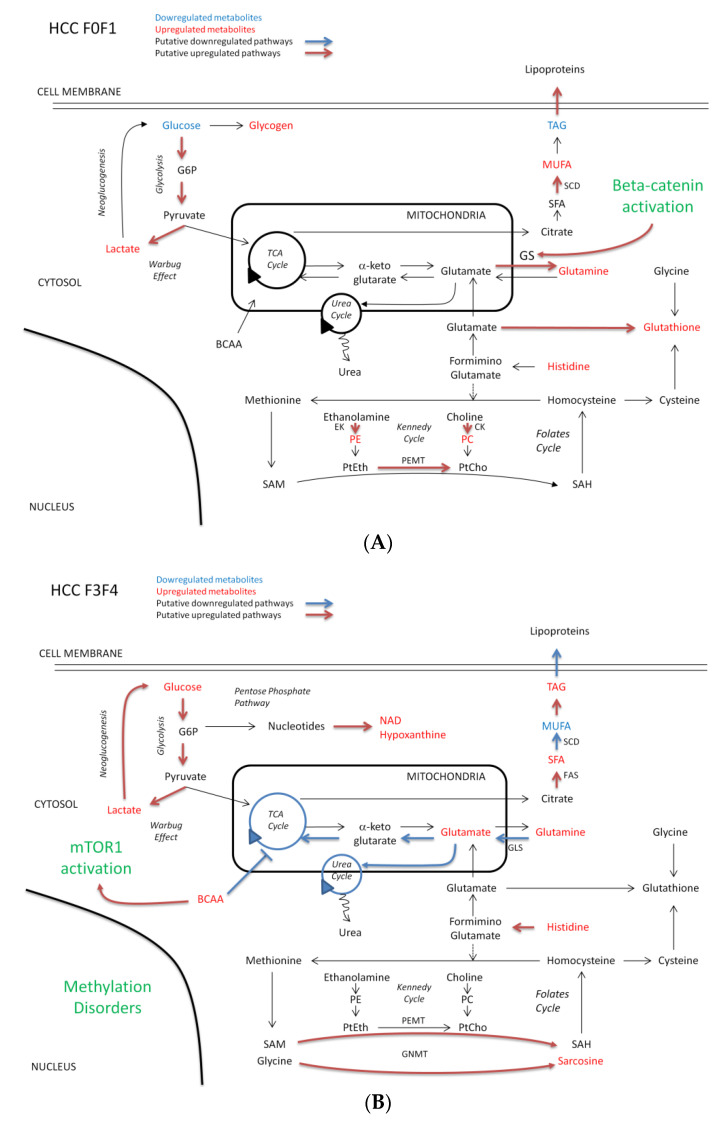
Discriminant metabolites identified and putative pathways involved in HCC according to the severity of fibrosis HCC-F0F1 (**A**) and HCC-F3F4 (**B**). Upregulated and downregulated metabolites are in red and blue, respectively. Upregulated and downregulated putative metabolic pathways are in red and blue, respectively. Abbreviations (in alphabetical order): BCAA, branched aminoacids; CK, choline kinase; EK, ethanolamine kinase, FAS, fatty acid synthetase; G6P, glucose-6-phosphate; GLS, glutaminase; GNMT, glycine N-methyltransferase; GS, glutamine synthetase; MUFA, monounsaturated fatty acids; NAD, nicotinamide adenine dinucleotide; PC, phosphocholine; PE, phosphoethanolamine; PEMT, phosphatidylethanolamine N-methyltransferase; PtCho, phosphatidylcholine; PtEth, phosphatidylethanolamine; SAH, S-adenosylhomocysteine; SAM, S-adenosylmethionine; SCD, stearoylcoA desaturase; SFA, saturated fatty acid; TAG, triacylglycerol; TCA, tricarboxylic acid.

Our study is a preliminary work suggesting different metabolic reprogramming and signaling pathways according to the severity of fibrosis (Figure 4A,B). However, NMR spectroscopy has its own weaknesses. It is not the optimal technique for lipidomics. Moreover, it is not a very accurate method for the identification of phospholipids, especially glycerophospholipids. Therefore, our results need confirmation and further exploration using mass spectrometry and transcriptomics.

## 4. Patients and Methods

### 4.1. Patients and Collection of Specimens

Liver tissue specimen were collected from the French Liver Biobank and selected (*n* = 56) according to the following criteria: (1) dysmetabolic liver disease without other associated causes, (2) stage of fibrosis defined with the METAVIR score by histological analysis, and (3) a balanced distribution of the stage of tumor differentiation (well or moderately). Tissue pairs with fibrosis at stage F2 were excluded (*n* = 9) from this analysis to obtain distinct and non-confusing groups. Indeed, previous studies demonstrated that fibrosis assessment using METAVIR classification was a source of disagreement with the lowest concordance observed for intermediate fibrosis grades (F1, F2, and F3 fibrosis) [47]. In addition, tissues from 5 patients undergoing hepatectomy at the University Hospital of Clermont-Ferrand were included with their written informed consent. Thus, 26 paired tissues of NAFLD-HCC F0F1 and 26-paired tissues of NAFLD-HCC F3F4 were analyzed (*n* = 52). The study was approved by the Ethic Committee Sud-Est VI Clermont-Ferrand (Agreement number AU887, 04/03/2011).

### 4.2. Histology

Tissues were fixed in 10% formalin and embedded in paraffin for light microscopy. Paraffin sections with a thickness of 5 µm were stained with hematoxylin and eosin method. HCC type and grades of differentiation (WHO Classification) were established. NTT was characterized by the presence or not of lesions of chronic hepatitis, fibrosis, steatosis, steato-hepatitis, and the METAVIR Score.

### 4.3. Sample Preparation for NMR-Spectroscopy

Tissue samples were processed to obtain aqueous and lipid extracts. A piece of tissue (0.075 g) was mixed with cold acetonitrile/water (1:1, *v*:*v*, 1.75 mL) and then homogenized over ice, using a polytron. Samples were centrifuged (17,000× *g*, 20 min, 4 °C), and the aqueous supernatant was centrifuged (17,000× *g*, 15 min, 4 °C) twice and dried to obtain the water-soluble fraction of liver extracts. The organic phase was dissolved in cold chloroform/methanol (2:1, *v*:*v*, 1.5 mL), homogenized, centrifuged (17,000× *g*, 20 min, 4 °C), and then filtered and dried to obtain the lipid phase. All reagents are conserved at 4 °C, and all experiments are conducted in ice. All dried samples were stored at −20 °C.

### 4.4. ^1^H-NMR Spectroscopy

Spectroscopy was performed at room temperature using a Brucker Advance 400 spectrometer (Brucker Corporation, Billerica, MA, USA) operating at 400.13 MHz. The dried extracts were rehydrated in 500 µL of D_2_O containing Phosphate Buffer (1%) (aqueous extract) or 500 µL chloroform-d/methanol-d (2:1, *v*:*v*) (lipid extract) in 5 mm diameter NMR tubes. For all samples, a one-dimensional ^1^H-NMR spectrum was acquired using a ZGPRESAT sequence with water signal suppression at low power and the following parameters: 8 µs −90° pulse length, 10-s relaxation delay, 10-parts per million (ppm) spectral width, 128 transients, and 32 K complex data points. Resonance assignment was carried out according to chemical shift values reported in the literature and the free access database Human Metabolome Database [48,49].

### 4.5. Data Processing

A line broadening factor of 0.3 Hz was applied to Free Induction Decay before Fourier transformation. ^1^H-NMR spectra were manually corrected for phase and baseline using MestReNova (Mestrelab Research chemistry software solutions, Santiago de Compostela, Spain). Peak referencing was done on the signal of creatine at 3.035 ppm for aqueous extracts and PtdCho at 3.26 ppm for lipid extracts. The spectra were binned into 0.02 ppm width data samples (from 0.5 to 9.5 ppm and from 0.5 to 7.0 ppm for the aqueous and lipid phases spectrum, respectively). All spectra were normalized to the total area under spectrum, after removing spectral regions containing solvent resonances: 4.69 to 5 ppm, 3.33 to 3.43 ppm, 7.20 to 7.80 ppm, corresponding to water, methanol, and chloroform, respectively.

### 4.6. Statistical Analysis

**Figure 5:**
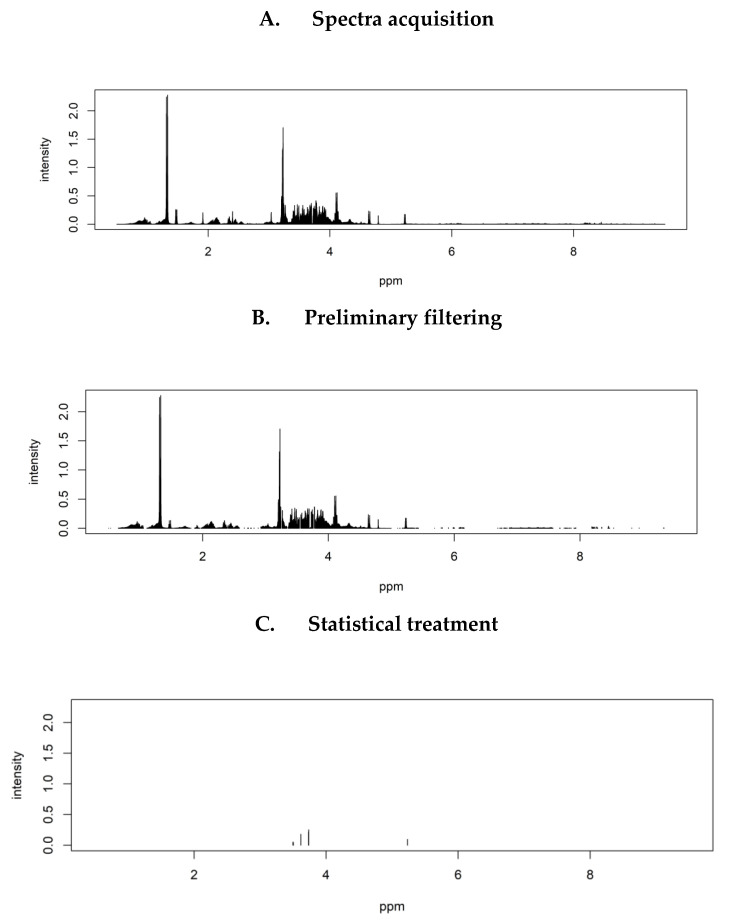
Complete workflow of the discrimination process: HCC-F0F1 compared to HCC-F3F4; Raw Nuclear Magnetic Resonance (NMR) aqueous spectra of HCC-F0F1 ≈ 4500 ion peaks (**A**); removal of technical artefacts, constant and redundant features = 1275 ion peaks (**B**); choice of the most discriminant metabolites in the aqueous phase by using Genetic Algorithm with Linear Discriminant Analysis = 5 ion peaks (minimum 45 selections), Final solution = 3 discriminant identified metabolites (**C**).

A pre-screening was proposed to remove useless features (ppm locations) according to discrimination: we removed technical artefacts, constant, and redundant features. We applied the latter two steps independently for each comparison (Figure 5).

A univariate analysis is not likely to highlight the best synergistic subset of features. Hence, a multivariable analysis using a combination of several metabolites is a more informative approach. However, after this pre-screening, it was impossible to test all feature subsets within a reasonable amount of time. We chose genetic algorithms (GAs) to perform the selection of subsets. GAs are optimization algorithms, based on the process of natural selection [50,51]. They provide approximate solutions to complex optimization problems. In a first step, a population of potential solutions is randomly generated. Then, this population evolves through the iterative application of mutation, cross-over and selection.

In our model, solutions were subsets of features. The mutation randomly altered each solution by addition, removal, or substitution of a feature. The cross-over randomly combines the features of two solutions. Selection is the only operator increasing the quality of solutions across generations. It relies on a fitness function quantifying the solution quality. A Linear Discriminant Analysis (LDA) was applied on each solution [52]. To avoid over-fitting, a two-fold cross-validation was used to evaluate the accuracy. The fitness function uses this accuracy penalized by the subset size to favor parsimonious solutions. For this purpose, we chose 10 as the maximal size for subsets. The GA was run 10 times, and the solutions obtained on the last generations were evaluated by the average cross-validated LDA accuracy. In order to identify the most interesting features, we used the frequency of selection of each feature in the final generations (Figure 5). Indeed, the more frequently a feature is selected to survive across generations, the more likely it is to play its part in discrimination. The value of the frequency threshold has been set using “random” GAs without any learning step.

## 5. Conclusions

Two metabolomics phenotypes of human HCC developed in NAFLD according to the degree of fibrosis have been revealed by proton NMR spectroscopy in our study. We highlighted the impact of the underlying pathology on metabolic reprogramming of the tumor. Except the common “Warburg effect”, the two groups of NAFLD-HCC exhibited distinct metabolomics alterations. HCC-F0F1 displayed abnormal levels of Plp derivatives and an increased content of Gln suggesting an overexpression of choline kinase and GS, respectively. In contrast, HCC-F3F4 exhibited an increased amount of BCAA implying a possible activation of the mTOR pathway and an increased content of glucose suggesting an enhanced neoglucogenesis.

The proposed metabolic signature might help to understand the specific molecular mechanism of NAFLD-HCC according to fibrosis level. Our results may lead to the development of new screening tools in NAFLD, especially in patients with no or mild fibrosis. Moreover, as a surrogate for tumor liver biopsy, MRI-based metabolic imaging may become relevant to discriminate subtypes of NAFLD-HCC according to the severity of fibrosis. Finally, NAFLD-HCC patients could be treated depending on their metabolomics signatures. Oncology care should be directed towards personalized therapies in the future.

## Figures and Tables

**Table 1:** Patients’ characteristics.

		**Group F0F1** ***N* = 26**	**Group F3F4** ***N* = 26**	***p*** **-Value**
**PARAMETERS**				
Gender (M:F)		21:5	24:2	ns
Age in years (mean ± SD)		69.9 ± 10.7	70.5 ± 5.9	ns
CLINICAL AND BIOLOGICAL DATA				
Body Mass Index (missing data *n* = 3)	Normal	4	4	ns
	Overweight	11	10	ns
	Obesity	10	10	ns
Diabetes (missing data *n* = 5)	Yes	16	19	ns
	No	7	5	ns
Tobacco (missing data *n* = 6)	Yes	7	11	ns
	No	16	12	ns
Blood Alpha-Foeto-Protein (missing data *n* = 4)	<20ng/mL	18	23	ns
	20–200ng/mL	2	0	ns
	200–1000ng/mL	1	1	ns
	>1000ng/mL	2	1	ns
HISTOLOGICAL DATA				
Degree of steatosis in NTT	No	5	1	ns
	Low (5–33%)	5	6	ns
	Moderate (33–66%)	11	14	ns
	Severe (>66%)	5	5	ns
Tumor Differentiation (WHO) (missing data *n* = 1)	Well	11	12	ns
	Moderate	14	12	ns
	Poor	1	1	ns

Significant difference: *p*-Value < 0.05 (Student Test, Fisher Test, Chi2 Test); ns: No significant differences between the 2 groups.

**Table 2:** List of discriminant metabolites identified with ^1^H-NMR spectroscopy and corresponding ppms.

	**Metabolites**	**Abbreviations**	**Chemical Shifts (ppm)**
**Aqueous** **Phase**			
**Carbohydrates/TCA cycle derivatives**	Lactate	Lac	1.31–1.33/4.10–4.11
	Glucose	Glc	3.39/3.46/3.51/3.75/4.63/5.22
	Glycogen	Gly	5.38–5.43
**Amino Acids and derivatives**	Glutamine	Gln	2.14/2.44
	Glutamate	Glu	2.04/2.34
	Glutathione	GSx	2.15/2.54/2.97/3.78
	Leucine	Leu	0.93–0.97
	Isoleucine	Isoleu	0.93–0.97
	Valine	Val	1.02-1.04/2.26
	Histidine	His	7.07–7.11
	Sarcosine	Sar	2.70–2.73
**Nucleotides derivatives**	Hypoxanthine		8.20
	Nicotinamide Adenine Dinucleotide	NAD	9.33
**Vitamins**	Ascorbic acid	Asc A	4.50
**Phospholipids Derivatives**	Phosphocholine	PC	3.22
	Choline derivatives		3.62–3.68
**Lipid Phase**			
**Phospholipids derivatives**	Phosphoethanolamine	PE	3.05–3.07/3.13
**Cholesterol**	Total cholesterol	TChol	0.69/0.93–0.94/1.01/1.52–1.54/2.19/2.25/3.49/3.57/3.89
	Free cholesterol	FChol	0.94/1.07/1.50/1.79/2.22/3.45/3.48/3.57
**Fatty acids**	Saturated FA (CH_2_)n	SFA	1.24–1.44
	Monounsaturated FA –CH_2_CH=	MUFA	2.02–2.12
**Triacylglycerides**	TAG	TAG	4.14–4.34 5.26

## Data Availability

Data is contained within the article or supplementary material.

## Supplementary Materials

The following are available online at https://www.mdpi.com/2218-1989/11/1/54/s1, Figure S1: Distribution of the size of the largest nodule according to the severity of fibrosis, Figure S2: Recurrence free survival (2A) and overall survival (2B) according to the severity of fibrosis, Figure S3: ROC (Receiver Operating Characteristic) curves of each subset of significant metabolites identified in aqueous (3A) and lipid phases (3B) for the discrimination of HCC from NTT in F0F1 fibrosis, Figure S4: ROC curves of each subset of significant metabolites identified in aqueous (4A) and lipid phases (4B) for the discrimination of HCC from NTT in F3F4 fibrosis, Figure S5: ROC curves of each subset of significant metabolites identified in aqueous (5A) and lipid phases (5B) for the discrimination of HCC-F3F4 from HCC-F0F1, Table S1: Discriminant metabolites between HCC-F0F1 versus NTT-F0F1 according to the number of selections in the aqueous and lipid phases. Upregulated (red) and down-regulated (blue) metabolites in tumors, Table S2: Discriminant metabolites between HCC-F3F4 versus NTT-F3F4 according to the number of selections in the aqueous and lipid phases. Upregulated (red) and down-regulated (blue) metabolites in tumors, Table S3: Discriminant metabolites between HCC-F0F1 versus HCC-F3F4 according to the number of selections in the aqueous and lipid phases. Upregulated (red) and down-regulated (blue) metabolites in HCC-F0F1.

## Abbreviations

HCC	hepatocellular carcinoma
NAFLD	non-alcoholic fatty liver disease
MS	metabolic syndrome
MAFLD	metabolic associated fatty liver disease
NAFLD-HCC	NAFLD associated HCC
NMR	nuclear magnetic resonance
NTT	non-tumoral tissue
ppm	parts per million
FA	fatty acids
AA	Aminoacids
Plp	Phospholipids
Lac	Lactate
Glc	glucose
Gln	glutamine
GS	glutamine synthase
His	histidine
GSx	glutathione
Asc A	ascorbic acid
PC	phosphocholine
PtdCho	Phosphatidylcholine
CK	choline kinase
MUFA	monounsaturated fatty acids
TCho	total cholesterol
AUC	area under curve
BCAA	branched chain aminoacids
Val	Valine
Leu	Leucine
IsoLeu	Isoleucine
Sar	Sarcosine
GNMT	Glycine N-methyltransferase
NAD	nicotinamide adenine dinucleotide
SFA	saturated fatty acids
DNL	de novo lipogenesis
FASN	fatty acid synthase
SCD	stearoyl coA desaturase
FCho	free cholesterol
TAG	Triacylglycerol
PE	phosphoethanolamine
PET	positron-emission tomography
FDG	fluorodeoxyglucose
ROS	reactive oxygen species
TCA	tricarboxylic acid
AAA	aromatic aminoacid
Gly	Glycine
THF	tetrahydrofolate
CDP PtdEth	cytidine diphosphate phosphatidylethanolamine
PEMT	phosphatidylethanolamine N-methyltransferase
MestReNova	Mestrelab Research chemistry software solutions
GA LDA ROC	Genetic Algorithm Linear Discriminant Analysis Receiver Operating Characteristic
