# Supplementary material for: Two Metabolomics Phenotypes of Human Hepatocellular Carcinoma in Non-Alcoholic Fatty Liver Disease According to Fibrosis Severity
Source: Metabolites. 2021 Jan 14;11(1):54. doi: 10.3390/metabo11010054 (PMC7830343; doi:10.3390/metabo11010054)
Supplement: Supplementary file 1 [file metabolites-11-00054-s001.pdf]

**Supplementary Materials**

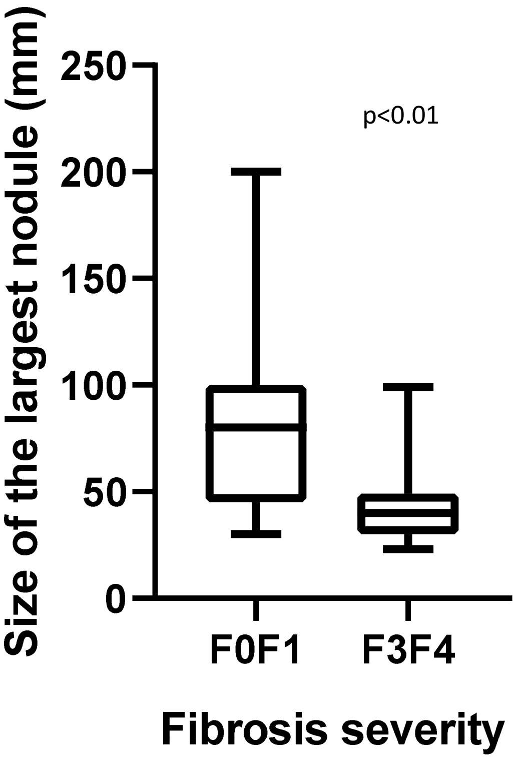


**Figure S1.** Distribution of the size of the largest nodule according to the severity of fibrosis (n=52).

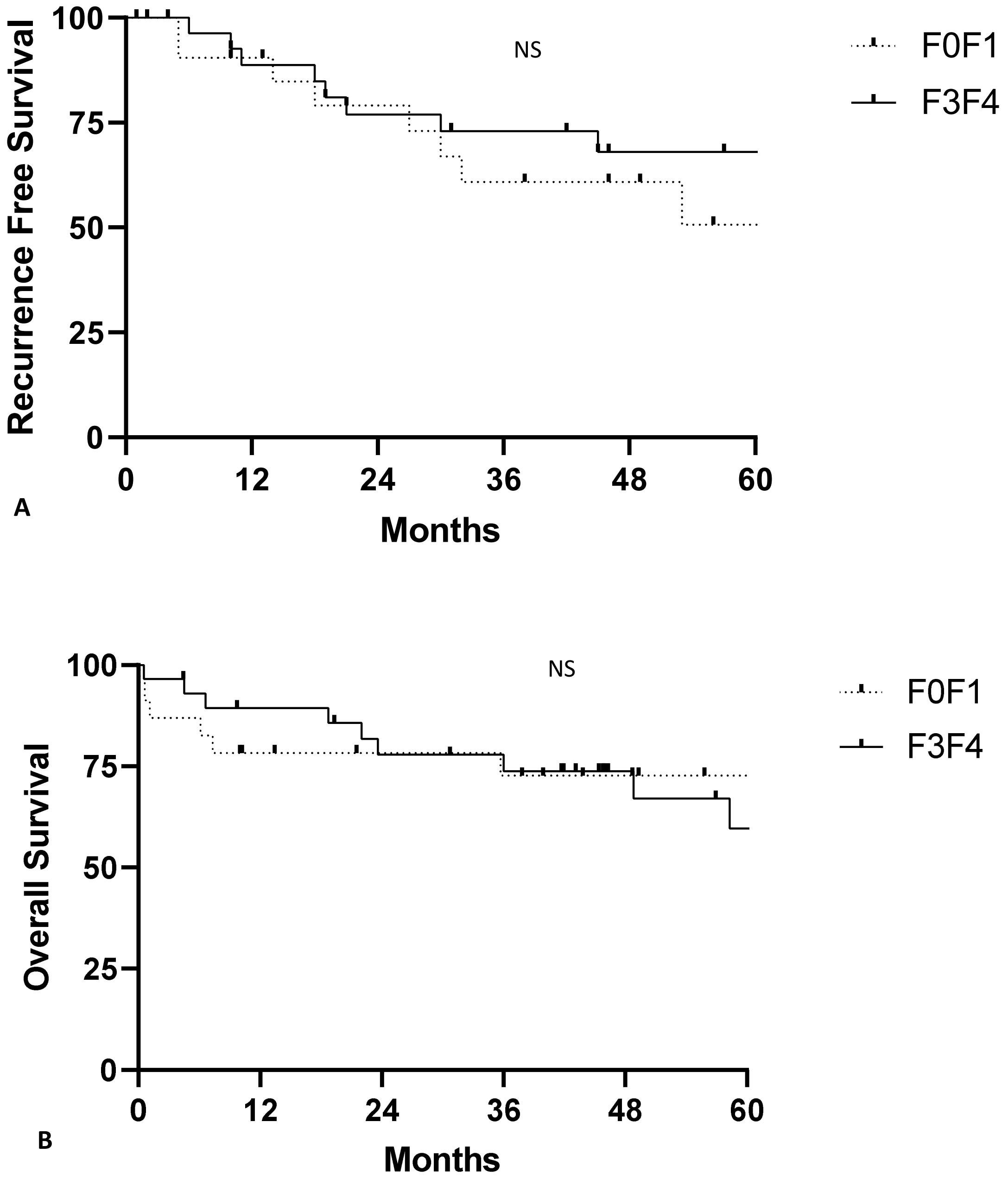


**Figure S2.** Recurrence free survival (**A**) and overall survival (**B**) according to the severity of fibrosis (n=52).

**Table 1.** Discriminant metabolites between HCC-F0F1 *versus* NTT-F0F1 according to the number of selections in the aqueous and lipid phases. Upregulated (red) and down-regulated (blue) metabolites in tumors.

| Chemical shift (ppm) | Metabolites | Selections |
|---|---|---|
| | **Aqueous Phase** (threshold: >47 selections) | |
| 1.31 | Lactate | 441 |
| 2.14 | Glutamine | 79 |
| 2.43 | | 53 |
| 2.56 | Glutathione | 72 |
| 3.22 | Phosphocholine | 48 |
| 3.62 | Choline Derivatives | 82 |
| 3.65 | | 76 |
| 3.66 | | 488 |
| 3.67 | | 60 |
| 3.68 | | 70 |
| 4.50 | Ascorbic Acid | 57 |
| 7.11 | Histidine | 54 |
| 7.85 | | 49 |
| 3.42 | Glucose | 50 |
| 5.43 | Glycogen | 104 |
| | **Lipid Phase** (threshold: >39 selections) | |
| 1.52-1.54 | Total Cholesterol | 81 |
| 2.25 | | 111 |
| 3.49 | | 67 |
| 2.05 | MUFA –$CH_2$CH= | 1339 |

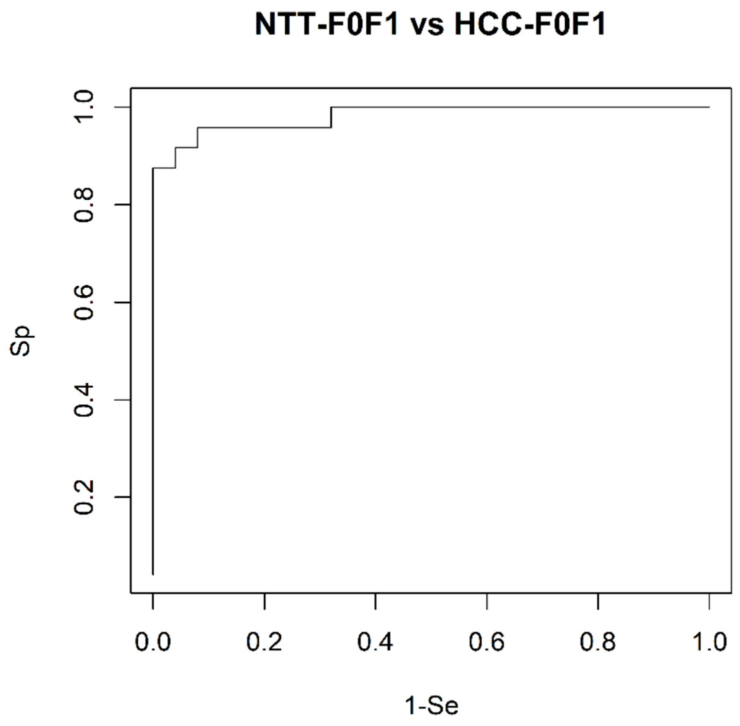


**A**

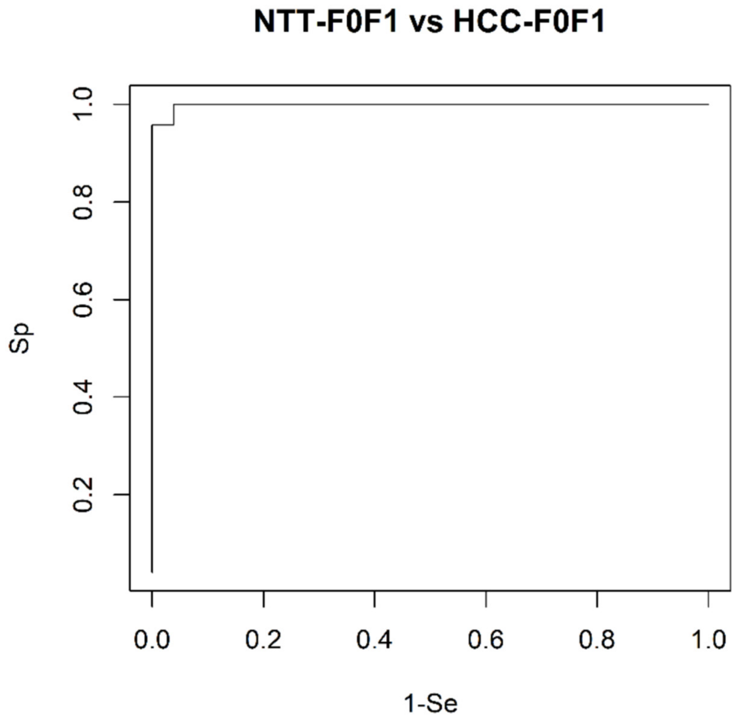


**B**

**Figure S3.** ROC curves of each subset of significant metabolites identified in aqueous (**A**) and lipid phases (**B**) for the discrimination of HCC from NTT in F0F1 fibrosis.

**Table S2.** Discriminant metabolites between HCC-F3F4 *versus* NTT-F3F4 according to the number of selections in the aqueous and lipid phases. Upregulated (red) and down-regulated (blue) metabolites in tumors.

| Chemical shift (ppm) | Metabolites | Selections |
|---|---|---|
| **Aqueous Phase** (threshold: >45 selections) | | |
| 0.97 | BCAA | 49 |
| 1.02 | | 46 |
| 1.31-1.32 | Lactate | 94 |
| 4.10-4.11 | | 71 |
| 2.06-2.07 | Glutamate | 50 |
| 2.38 | | 48 |
| 2.12-2.14 | Glutamine | 89 |
| 2.42-2.43 | | 48 |
| 2.73 | Sarcosine | 91 |
| 3.63 | | 106 |
| 8.20 | Hypoxanthine | 87 |
| 9.33 | NAD | 67 |
| 3.67 | Choline derivatives | 51 |
| 3.82 | Glucose | 56 |
| 4.60 | | 49 |
| 6.98 | Histidine | 64 |
| 7.10 | | 124 |
| **Lipid Phase** (threshold: >21 selections) | | |
| 1.41-1.43 | SFA $(CH_2)n$ | 606 |
| 1.83 | Total Cholesterol | 98 |
| 2.22 | Free Cholesterol | 84 |
| 2.06 | MUFA $–CH_2CH=$ | 151 |

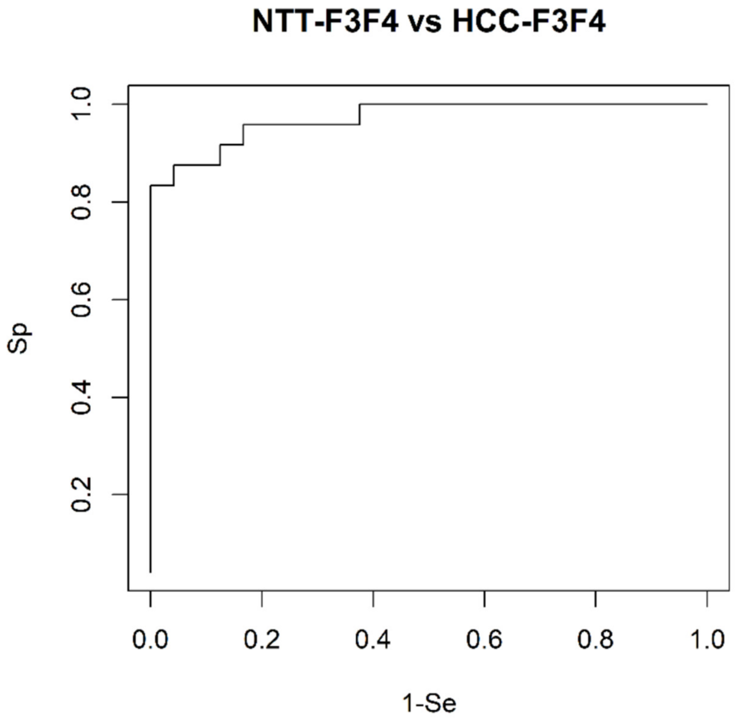


**A**

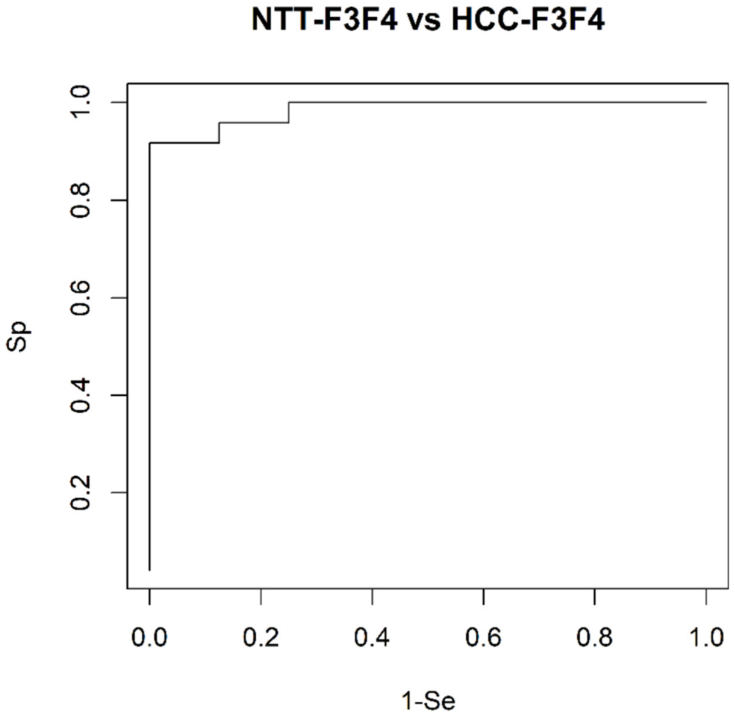


**B**

**Figure S4**: ROC curves of each subset of significant metabolites identified in aqueous (**A**) and lipid phases (**B**) for the discrimination of HCC from NTT in F3F4 fibrosis.

**Table S3.** Discriminant metabolites between HCC-F0F1 *versus* HCC-F3F4 according to the number of selections in the aqueous and lipid phases. Upregulated (red) and down-regulated (blue) metabolites in HCC-F0F1.

| Chemical Shift (ppm) | Metabolites | Selections |
|---|---|---|
| **Aqueous Phase** (threshold: >45 selections) | | |
| 3.50 | Glucose | 2756 |
| 3.74 | | 55 |
| 5.23 | | 61 |
| 3.62 | Choline derivatives | 2086 |
| **Lipid Phase** (threshold: >50 selections) | | |
| 2.11 | MUFA | 61 |
| 5.46 | | 64 |
| 3.05-3.07 | Phosphoethanolamine | 374 |
| 4.12-4.22 | TAG | 531 |

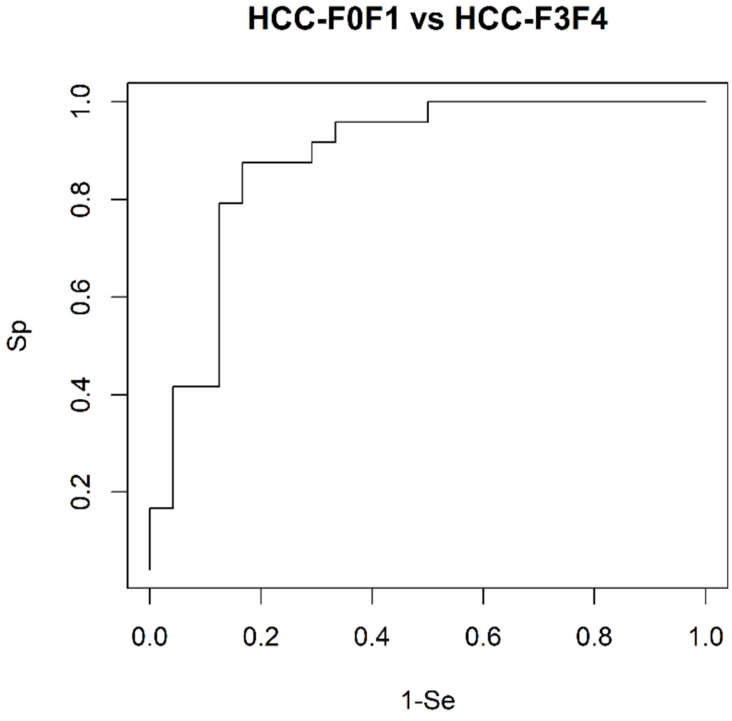


**A**

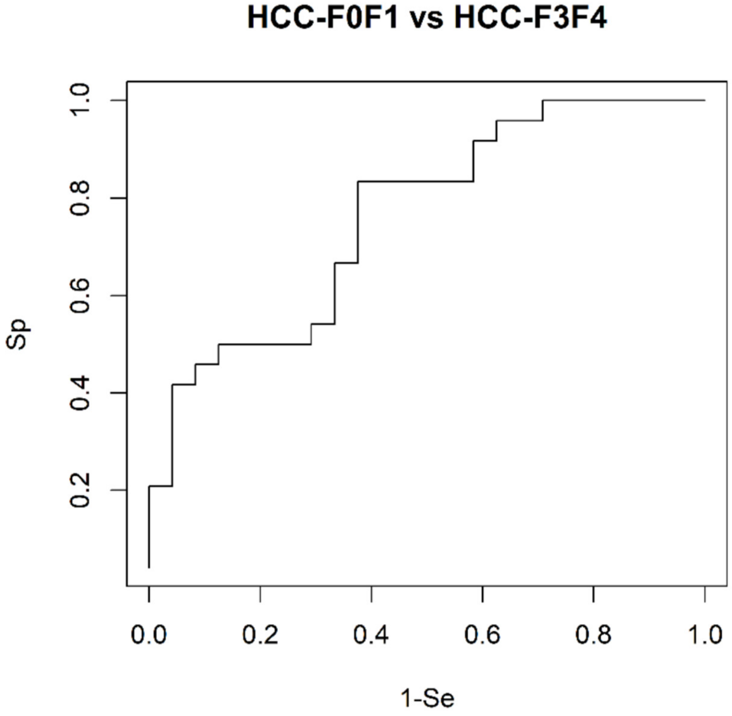


**B**

**Figure S5:** ROC curves of each subset of significant metabolites identified in aqueous (**A**) and lipid phases (**B**) for the discrimination of HCC-F3F4 from HCC-F0F1.
